# Manipulation of the immune system by non-small cell lung cancer and possible therapeutic interference

**DOI:** 10.20517/cdr.2020.40

**Published:** 2020-09-12

**Authors:** Helmut H. Popper

**Affiliations:** Medical University of Graz, Research Unit Molecular Lung & Pleura Pathology, Institute of Pathology, Neue Stiftingtalstrasse 6A, Graz 8036, Austria.

**Keywords:** Programmed death 1 - programmed death ligand 1, lymphocyte subpopulation, innate immune cells, soluble mediators of immune tolerance, phagocytosis inhibitors

## Abstract

Pulmonary carcinomas have developed mechanisms by which they escape the attack of immune cells. Immune checkpoint molecules programmed death 1 - programmed death ligand 1 (PD1-PDL1) and the cytotoxic T-lymphocyte antigen 4 system have gained attention. The expression of PDL1 by tumor cells causes immune tolerance, and further influences the microenvironment via orchestration by cytokines. Therapy with PDL1 antibodies could restore the cytotoxicity of T-lymphocytes towards tumor cells. Many patients will respond to this treatment. However, resistance mechanisms will counteract this therapy. New investigations have identified additional immune checkpoint inhibitors such as lymphocyte activation gene 3 and T cell immunoglobulin and mucin-domain containing-3. Tumor cells also induce tolerance by manipulating cells of the innate immune system. Macrophages are polarized to tumor-friendly M2, neutrophils into N2 types, and dendritic cells and myeloid suppressor cells are switched to assist tumor cells. Regulatory T cells enter the tumor microenvironment and signal tolerance to cytotoxic cells, inhibiting the influx of NK cells. Soluble mediators either released by tumor cells or cells of the tumor stroma induce immune tolerance, examples including tryptophan and indolamine dioxygenases, arginine and adenosine. Treatment options to counteract these molecules are currently being tested. The tumor stroma has been classified as immune-inflamed, immune-excluded, and immune-desert types. The latter might be switched to an inflamed type by induction of tertiary lymph follicles. Dendritic cells and macrophages normally phagocytose tumor antigens, but inhibitors of phagocytosis can block this. Interference with these molecules is another option for re-establishing the cytotoxic action of the immune system against tumor cells. In this review we will discuss these aspects with a special emphasis on non-small cell lung cancer.

## Introduction

Immunotherapy was first applied to melanomas, which is one of the malignancies with the highest mutational burden. But shortly after it was reported that non-small cell carcinoma (NSCLC) can be targeted by antibodies for immune checkpoint inhibitors. A major breakthrough in establishing this type of therapy was the discovery of immunomodulation of T cells response through immune checkpoints inducing immune evasion of cancer cells. Cytotoxic T-lymphocyte antigen 4 (CTLA-4) was not only found in dendritic cells and regulatory T cells (Tregs) in lymph nodes, but also circulating in the blood, inducing apoptosis and cell death of cytotoxic T-lymphocytes^[1]^. Programmed cell death 1 (PD1) and its ligand PDL1 was found on tumor cells as well as on lymphocytes inducing immune tolerance^[2]^. Based on these findings, antibodies for PDL1 and PD1 were tested for their ability to interfere with this mechanism of immune tolerance against tumor antigens. Important efforts are under way and have focused on this new treatment modality^[2-10]^. At present, several pharmaceutical companies have developed checkpoint inhibitors (humanized monoclonal antibodies) for the treatment of NSCLC patients and several phase II and III studies have been performed. The FDA and EMA have recently approved three of these drugs for treatment of NSCLC. Whereas some drugs are selectively approved for squamous cell carcinomas (SCC), others may be used in all NSCLC^[9,11-14]^.

## Immunotherapy of NSCLC

**Figure 1:**
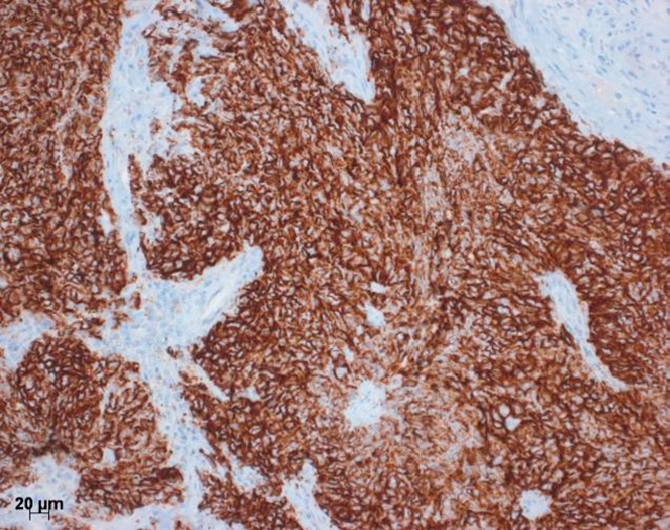
Immunohistochemistry for PDL1. Here a squamous cell carcinoma to almost 100% positive for PDL1. This would be an ideal candidate for the PDL1 antibody therapy. Bar 20 µm. PDL1: programmed death ligand 1

Like other solid tumors, pulmonary carcinomas have developed several mechanisms by which they escape the attack of cytotoxic immune cells. Some of these mechanisms have recently gained special attention: the programmed death 1 - programmed death ligand 1 and 2 (PDL1/2-PD1) and the CTLA4 system. Tumor cells as well as lymphocytes can express ligands for PD1^[4]^. Through this process, they interact with surface molecules on CD8^+^ T cells causing apoptosis, and furthermore influence the microenvironment via orchestration by cytokines, which all together cause immune tolerance. Therapy using antibodies against PDL1 have shown significant improvement experimentally as well as in clinical studies to restore the cytotoxic attack of T-lymphocytes towards tumor cells in several solid malignancies^[3,6,10]^. Parallel to targeting PDL1 by anti-PDL1 antibodies, antibodies against the receptor PD1 have also been created and used experimentally with success^[2,4,5,7,8]^. In some clinical trials, specific immunohistochemistry tests for the expression of PD1 and PDL1 were used to select those patients, who might best respond to the antibody-based therapy. A strong staining in at least 5% of tumor cells and/or lymphocytes - or 50% in another trial^[15]^- was regarded as a positive result and predictive for outcome [Figure 1]. The clinical data of these trials showed that majority of patients are detected by this simple stain. This however, created problems: In the clinical studies each company used a different antibody for PDL1 immunohistochemistry, and even more different staining platforms were used. This created problems for the pathology laboratories: purchasing all antibodies and also the staining platforms is impossible or would significantly increase the price for the tests. Finally, in a multi-institutional study, all antibodies and platforms were investigated, and three antibodies showed a similar staining performance^[16]^. But soon after, other anomalies emerged: patients who do not respond to treatment although being positive for PD1/PDL1 exist, as well as patients who do respond despite being negative or low positive for these immunohistochemical tests^[17-19]^.

New research has addressed this issue. Even if the reaction of cytotoxic lymphocytes (Tcyt) against tumor cells is restored, that does not mean the Tcyt will recognize tumor cells, because the tumor cells do not express many neoantigens^[20]^. In addition, some neoantigens might be low immunogenic, i.e., the epitopes are recognized, but elicit only a mild reaction with low affinity antibodies, or antibodies, which do not induce a combined cytotoxic reaction together with T cells^[21-23]^. The evaluation of mutational burden (MTB) was therefore proposed as an additional test to select those patients who will benefit from immunotherapy^[24,25]^. However, MTB alone was not sufficient to identify patients profiting by immunotherapy: a combination of PDL1 expression on tumor cells and a high MTB better characterized these patients^[24,26,27]^. Even these combined tests do not fully cover all patients and resistance to this treatment is possible. Processing of neoantigens for presentation to Tcyt also requires the cooperation of molecules from the major histocompatibility complex (MHC). Tumor antigens can be non- or low-immunogenic, for example, aberrant expressed/overexpressed proteins from trophoblast, cancer-testis proteins (an example is the MAGE cluster; some of these have been used for vaccination). These antigens usually bind to MHC-I molecules and most often only elicit a transient immune reaction^[20,28,29]^. Recently, a report was published showing that the MHC-I complex might be digested by an autophagy mechanism, therefore inhibiting the procession of an antigen^[30]^. Mutated neoantigens are presently those eliciting a good and lasting immune reaction; examples are proteins derived from mutated TP53 gene in cigarette smokers.

**Figure 2:**
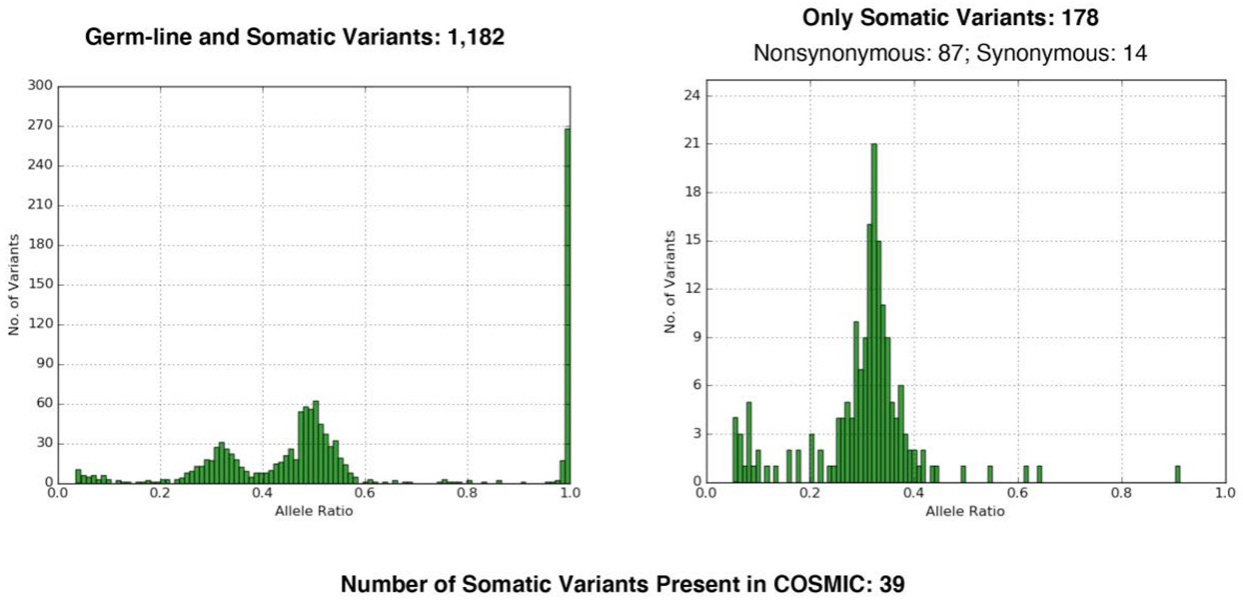
Tumor mutational burden. On the left side germline and somatic mutations are shown for the normal control tissue, to the right high numbers of mutations are seen in the tumor. Such a finding might help to decide for an immunotherapy

To recognize a tumor neoantigen as non-self and immunogenic, MTB was established as a method to analyze the number of mutations in a given tumor^[27,31]^. Different types of next generation sequencing platforms can be used: whole exome sequencing, whole genome, or sequencing of a cancer gene panel. Cutoffs have been established to separate high- from low-mutational burden [Figure 2]. One problem in using MTB is the necessity to have normal control tissue from the patient.

**Figure 3:**
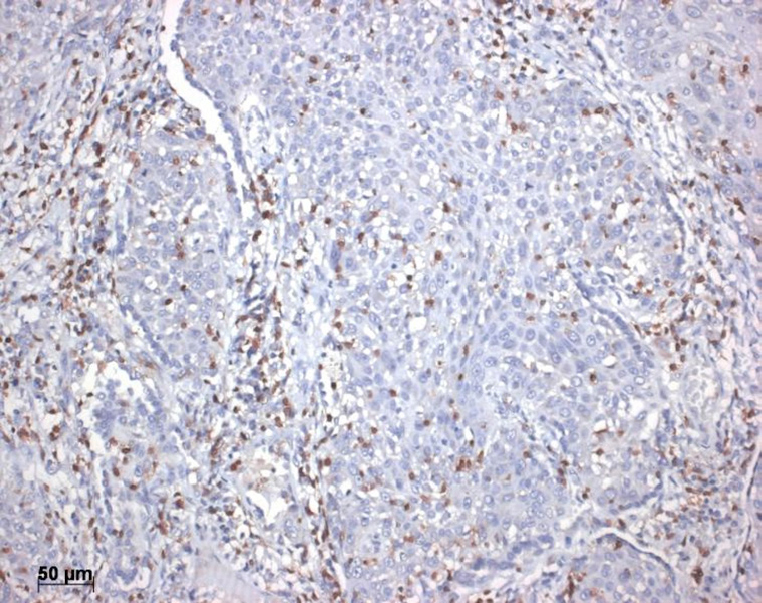
CD8+ lymphocytes infiltrating this solid adenocarcinoma. Bar 50 µm

New results were reported from the analysis of T-cell receptors on Tcyt: The recognition of tumor neoantigens could be evaluated, and this method showed a good correlation with response to immunotherapy^[20,26,32-34]^. In another approach, tumor infiltrating lymphocytes were screened. If there was a high number of tumor-infiltrating CD8^+^ lymphocytes, in most cases a good response to immunotherapy was seen [Figure 3]. Even more sensitive was the analysis of these lymphocytes: the best response to therapy and increased survival was seen in those cases where the lymphocytes predominantly expressed CD8^+^ together with TCF7^+20^. Polymorphisms of the HLA genes might also influence the immune reaction against cancer neoantigens. The presence of HLAB44 corresponded favorable with immunotherapy resulting in prolonged survival^[35,36]^. On the contrary, patients with some germline mutations on HLA-I loci have shown a reduced response to immunotherapy^[37]^.

Before we proceed into new protocols of how to restore the immune reaction against cancer cells, we need to recapitulate the principals of an immune reaction This will not be an extensive review on the subject but a brief and simplified presentation, helping to understand the following paragraphs.

Tumor cells are either destroyed by cells of the innate or specific immune system, or die due to hypoxia. These debris need to be removed by phagocytosis; macrophages are the main population, which will take up the debris and process the neoantigens from tumor cells. Macrophages subsequently present these processed antigen fragments to dendritic cells or directly to lymphocytes/NK cells. In conjunction with antigens, costimulatory and MHC molecules have to be present to stimulate an immune reaction. Immune check point molecules will next decide if there is tolerance for these antigens (self-antigen), or if this antigen-bearing cells should be eliminated. Different cells of the immune system will cooperate during an immune reaction, including: cytotoxic T cells either CD8^+^ or CD4^+^, CD20^+^ B cells, NK cells, but also N1 neutrophils, and M1 macrophages. Along all these steps, interventions are possible, which we will cover in the following paragraphs.

**Figure 4:**
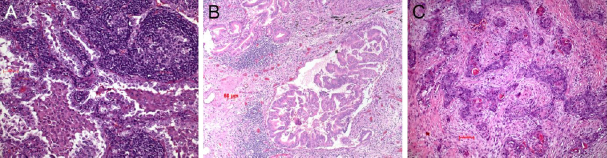
Immune inflamed adenocarcinoma. Note the intimate association of lymphocytes with the carcinoma cells (A); immune excluded type, an acinar adenocarcinoma. The lymphocytes are seen respecting the tumor boundaries (B); immune desert type of carcinoma, here a squamous cell carcinoma (C). Bars 50 and 100 µm

Another approach was to analyze the microenvironment (TME) of the tumor in more detail. Three types were recognized so far: the immune inflamed, the immune excluded, and the immune desert tumor [Figure 4]^[38]^. In the inflamed type there are CD4^+^ and CD8^+^ T cells within the tumor stroma and between tumor cells. These lymphocytes and tumor cells usually express PD1/PDL1 (besides other regulatory molecules; see below). In the excluded type there are different immune cells in the surrounding stroma but not within the tumor. In this case most often the WNT-β catenin pathway and TGF is activated^[39,40]^. Lymphocytes are absent or scarce in the desert type. These patients do not respond to immunotherapy^[20,38]^. In this type, molecules within the circulation or expressed on endothelia inhibit the extravasation of immune cells; there is ongoing research to explore these mechanisms and find possibilities to interfere with the blockade^[41-44]^. We will come back to this later on.

Although PD1-PDL1 has been proven as targetable molecules within the immune regulatory system, it is well known from non-neoplastic diseases, that there are many more ways the immune system can be shifted towards antigen tolerance or even lymphocyte exhaustion. Several of these regulatory mechanisms are also targetable. Subsequent development of resistance towards the PD1-PDL1 therapy might be due to upregulation of one of these immunomodulators. In addition, patients might have developed tolerance against their tumor cells not only by expression of PD1-PDL1, but also by other tolerance mechanisms - this could occur in patients who do not respond to anti-PDL1 therapy despite having PD1-PDL1 upregulated. Finally, in some cases tumor cells escape the immune cell attack by shedding or losing some of their neoantigens^[45]^.

## Other checkpoint inhibitors/regulators

The search for other molecules functioning as immune check-point controls has identified several new molecules, such as lymphocyte activation gene 3 (LAG3), T cell immunoglobulin and mucin-domain containing-3 (TIM3), tumor necrosis factor OX40 (CD134). While OX40 is a stimulating molecule, LAG3 and TIM3 are inhibitory. Treatment protocols have been developed (analogs and inhibitors) and are currently used in clinical studies usually in combination with PDL1 inhibition. Similar to PD1/PDL1 axis, LAG3 and TIM3 can also induce immunotolerance. TIM3 is suspected to even signal super-exhaustion to Tcyt^[46]^.

So far there are preliminary data on these molecules and OX40 does not seem to be very effective. There are conflicting reports on the combination of PDL1 and OX40 therapies: firstly, the expression of PDL1, OX40 and OX40L varies considerably among NSCLC tested, and secondly low expression of OX40 was associated with longer overall survival and better prognosis. Other studies also reported on OX40, GITR and 4-1BB, other receptors implicated in immune homeostasis. Although tumor regression was seen in experimental setting, this was associated with severe autoimmune events (so called cytokine storm) due to loss of Treg homeostasis^[47-50]^. Other regulatory molecules including LAG3 and TIM3 were studied in combination with PDL1 blockade. The most advanced involved LAG3, which results in the exhaustion of T cells. Several in-vivo approaches demonstrated a highly significant clinical benefit under dual blockade of PDL1 and LAG3, whereas the efficacy was very low in cases of single agent targeting^[51]^. Human tumor tissues showed co-expression of LAG3 and PD-1 in infiltrated lymphocytes. The ongoing clinical studies mainly used dual blockage of LAG3/PD-1, which demonstrated promising survival benefits and long duration of response rates. Combining immunotherapy of anti-LAG-3 and anti-PD-1 has shown exciting efficacy in fighting PD-1 resistance^[52,53]^. Clinical studies on the combination of PDL1 and T-cell Immunoglobulin- and Mucin-domain-containing molecule 3 (TIM3) are ongoing, however, the significance has not been proven so far. TIM3 is expressed on lymphocytes and seems to be a marker for super-exhaustion of these cells. If the combination therapy might overcome the resistance will be seen^[46,54]^. Primarily not much attention was given to cells of the innate immune system and cells cooperating with lymphocytes.

## Systems known to be able to induce immune tolerance towards foreign antigens

One of the most important cell types in this regard are antigen-presenting cells from the dendritic cell lineage. There are several populations, such as conventional dendritic cells, which promote immune attack by processing foreign antigens and presenting them to CD8^+^ T-lymphocytes. Others such as plasmocytoid and granulocytic dendritic cells cause immune tolerance by either cooperating with regulatory T cells or by inducing an inflammatory environment, which promotes tumor cell invasion, spread, and finally metastasis^[55,56]^. The function of Langerhans cells, another dendritic cell type, is entirely unknown, but most probably act against tumor antigens. We will come back to other aspects of dendritic cell function.

**Figure 5:**
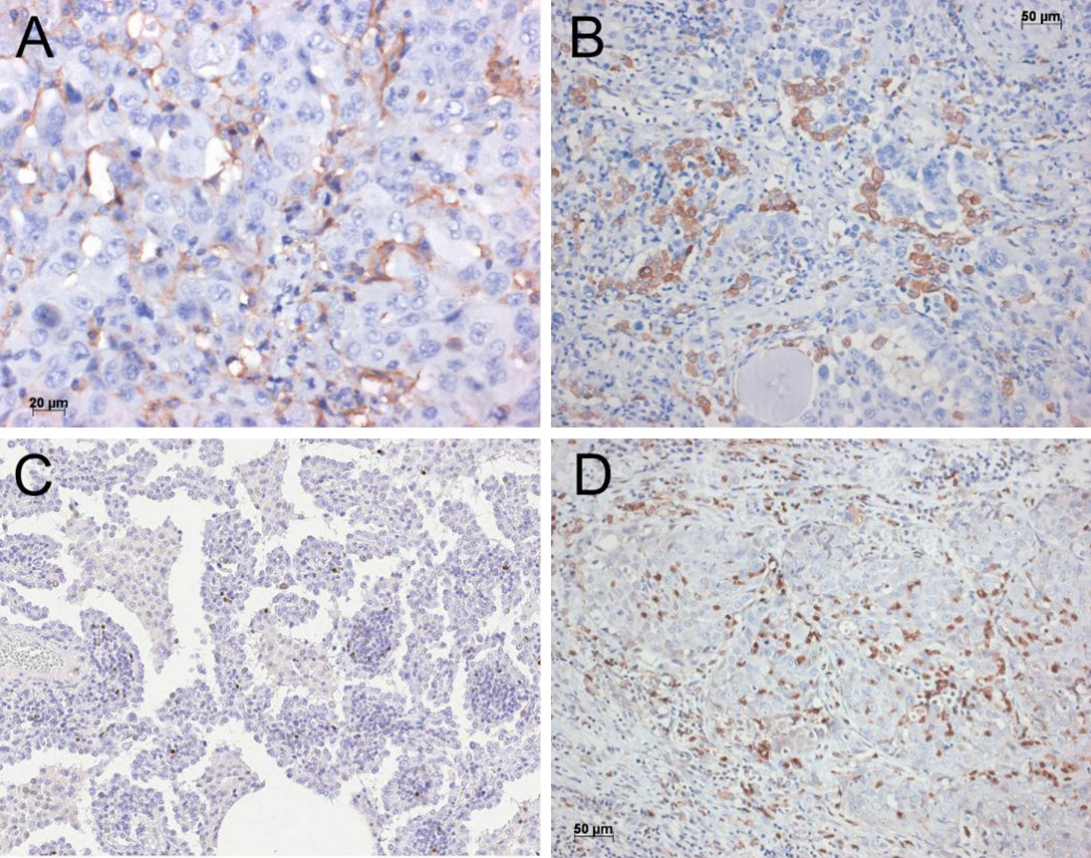
Plasmocytoid dendritic cells closely intermingled with the carcinoma cells (A); macrophages of M2 type are intimately associated with the adenocarcinoma cells. Immunohistochemistry for CD206 (B); increased numbers of regulatory T cells within the tumor stroma of this adenocarcinoma. Immunohistochemistry for FOXP3 (C); myeloid derived suppressor cells closely associated with cells of this squamous cell carcinoma. Immunohistochemistry for CD11b (D). Bars 20 and 50 µm, and magnification 200×

Plasmocytoid dendritic cells [Figure 5A] can also interact with monocytoid cells by promoting the differentiation of macrophages into the tumor-promoting M2 lineage^[57]^. Macrophages play a major role early on in carcinogenesis. Macrophages can induce angiogenesis, prepare and modulate stroma proteins in favor of invading tumor cells, thus promoting tumor growth, invasion, and metastasis^[58,59]^. On the contrary, M1 macrophages might inhibit tumor progression not only in the early phase but also during metastasis formation^[60,61]^.

There are additional pathways by which tumor cells can influence the differentiation of M0-macrophages into M2 lineage. Whereas activation of Notch signaling increased M1 macrophages, blocking of Notch induced an M2 response. Wang *et al*.^[60]^ showed that RBP-J-mediated Notch signaling regulates the M1 versus M2 polarization through SOCS3. Notch is inactivated by mutation in several lung carcinomas, however, the link with tumor-associated macrophages has not been studied so far. Tumor cells might use SOCS3 signaling to induce M2 macrophage polarization [Figure 5B]. Studies are on the way to influencing the differentiation of macrophages, as it is known that M2 macrophage polarization can be reverted^[62-64]^.

Regulatory T cells (T_reg_) can accumulate at the tumor site and block CD8^+^ T-lymphocytes and NK cells [Figure 5C] in their anti-tumor action as well as inhibit influx of the cells into the tumor^[65,66]^.

Myeloid-derived suppressor cells have been shown in pulmonary and squamous cell adenocarcinomas [Figure 5D]. These cells not only inhibit Tcyt, but also assist tumor cell metabolism under hypoxic condition^[67-70]^.

**Figure 6:**
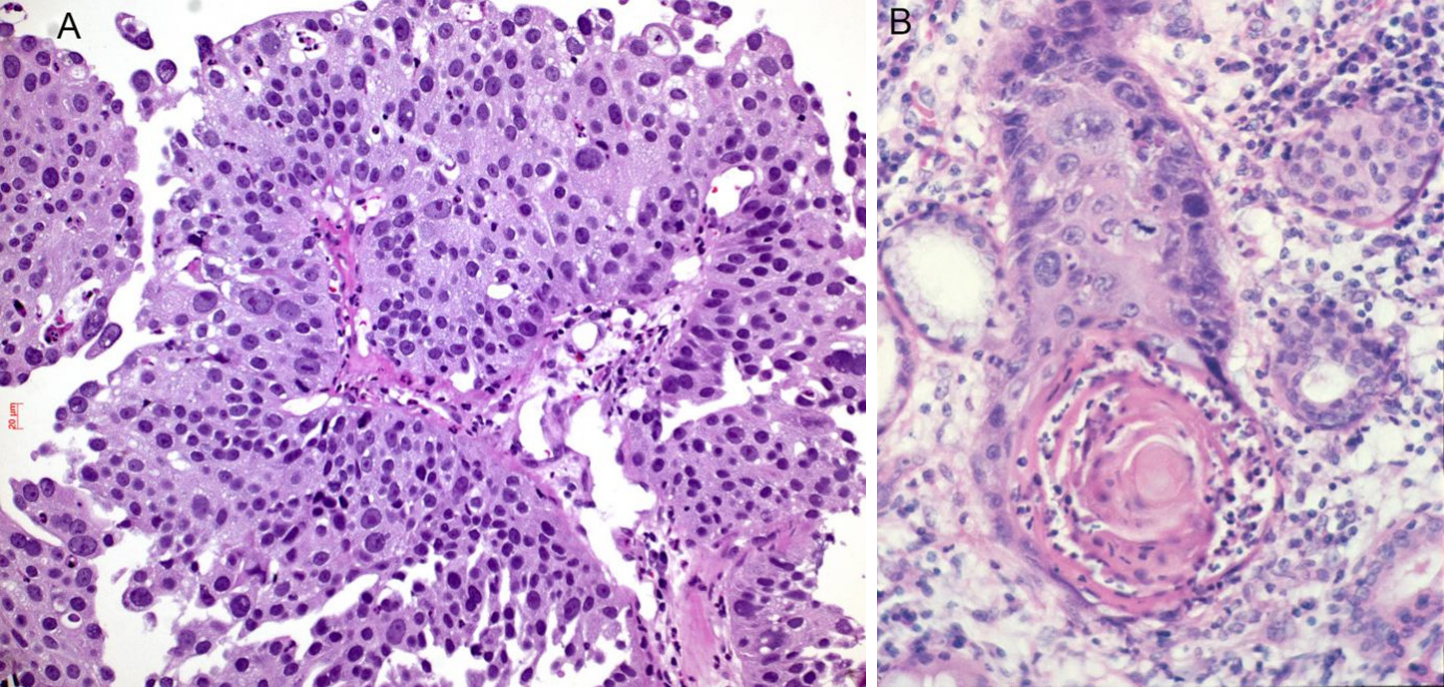
Infiltration of neutrophils in squamous cell carcinomas: neutrophils within the carcinoma (A), neutrophils concentrated in the keratin pearl (B). Magnification 200×

Neutrophils have recently gained recognition as a part of the innate immune system. Neutrophils can often be seen in squamous cell carcinomas, probably attracted by keratinized tumor cells and cell-free keratin, which can activate the complement cascade and in turn induce influx of neutrophils [Figure 6]. Moreover, there are two types of neutrophils: N1 and N2^[71-73]^. The N2 type seems to favor and promote lung cancer progression and escape of immune cell attack. These tumor-associated neutrophils interact with CD47, inhibiting phagocytosis by macrophages and increase inflammation. In patients refractory to PD1/PDL1 therapy, a myeloid-rich group of lung cancer cases was seen: in these cases, neutrophils outnumbered CD8^+^ and CD4^+^ lymphocytes and a neutrophil antagonizing strategy might help to overcome resistance to immunotherapy.

Autophagy is another mechanism associated with immune cell modulation, although this mechanism is presently not fully understood. Autophagy is seen in many lung carcinoma types. Increase of autophagy in cancer cells liberates nutrients, decreases the formation of reactive oxygen species, and aids in the clearance of misfolded proteins. This provides a survival advantage for cancer cells in the TME. However, immune cells also infiltrate the tumor environment and encounter hypoxia, resulting in hypoxia-induced autophagy. Due to the fact that autophagy is crucial for immune cell proliferation as well as for antigen presentation and T cell-mediated killing of tumor cells, anticancer treatment strategies based on autophagy modulation will need to consider the impact of autophagy on the immune system^[74]^. In melanomas, it has been shown that hypoxia leads to instability of gap-junctional CX43 and impairs melanoma cell killing by NK cells. Inhibition of autophagy by pharmacological approaches might restore NK cell mediated lysis of hypoxic melanoma cells^[75]^.

## Soluble mediators

News with a potential to interfere with immunotolerance signals from the tumor and the tumor-associated macrophages have been identified in metabolic studies. The amino acids tryptophan (trp) and arginine (arg), if present in sufficient concentration within the tumor MEV? can elicit immune tolerance. Trp is catabolized by tryptophan-2,3-dioxygenase or by indolamine-2,3-dioxygenase (IDO) to kynurenic and picolinic acid. Arg is metabolized predominantly by macrophages in two different ways: M1 macrophages metabolize Arg into nitric oxide and citrulline via nitric oxide synthase, whereas M2 macrophages will metabolize Arg into ornithine and urea via arginase. These latter metabolites will inhibit T cells and NK function^[76]^. A therapy using an arginase inhibitor showed promising results by inhibiting tumor growth, especially when combined with standard chemotherapy^[77]^. It was also shown that IDO also affects dendritic cells. Experimentally silencing IDO can create a robust anti-tumor immunity with activation of anti-tumor acting dendritic cells cooperating with cytotoxic T cells^[78]^. This could lead to the discovery of a new line of NSCLC immunotherapy.

**Figure 7:**
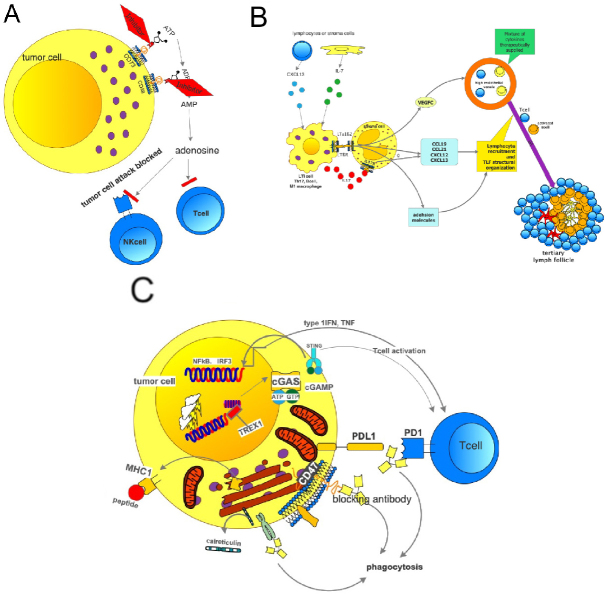
Mechanism of adenosine generation and its effects blocking T and NK cells. The generation of adenosine can be inhibited by either blocking the CD73 or CD38 exonucleases. Trials have been started (A); how tertiary lymph follicles can be formed. On the left side the natural mechanisms are shown. On top right the possible therapeutic intervention is shown (B); how to block the “no-eat-me” signal: a therapy using a blocking antibody for CD47 in combination with antiPDL1 will result in antigen processing by a macrophage, presentation of the antigen to cytotoxic T cells; as the PDL1 on expressing tumor cell is also blocked by an antiPDL1 antibody, the T lymphocyte can act and finally kill the tumor cell (C). PDL1: programmed death ligand 1

Another factor involved in resistance mechanism of immunotherapy is adenosine. Adenosine is created from ATP via ADP-AMP by the ectonucleotidases CD73 and CD38. Either tumor cells or macrophages can express these enzymes. Metabolites as well as arginine itself can induce immunotolerance in Tcyt^[79-85]^. As these amino acids and their enzymes are part of the immune machinery in inflammation, these are most likely involved in immune reactions of the inflamed and the excluded types and interference with these molecules can be an additional strategy to overcome immune tolerance to cancer cells. However, these amino acids are also necessary for T cell proliferation, which should not be blocked. Accumulation of adenosine is seen especially in hypoxic areas. Adenosine blocks NK cells by different mechanisms, such as cytolytic activity, expression of cytotoxic granules, and interferon g release^[86]^. Extracellular adenosine suppresses pro-inflammatory activities upon binding with adenosine receptors on the surface of various immune cells. In addition, signaling through adenosine receptors upregulates a number of anti-inflammatory molecules and immunoregulatory cells, leading to the establishment of a long-lasting immunosuppressive environment^[87]^. Treatment options are available to block CD73 but not for CD38 [Figure 7A]. Some treatment protocols use a combination of anti-PD1/PDL1. A CD73 inhibitor MEDI-9,447 influences myeloid and lymphocyte infiltrations in the TME: an increase of CD8^+^ lymphocytes and activated macrophages was noted. A combination with PD1 antibodies was also tested. Another study analyzed the concentrations of ATP and ADP in BAL fluid and found a significant increase in cancer patients, and in patients with metastatic disease. This study again showed a rationale for the blockade of the CD73-CD38-arginine system. Finally, a study also concluded that blocking the adenosine receptor A2AR together with anti-PD1 was effective in the treatment of metastatic and residual disease^[88-91]^.

Adenosine receptors have another function which was recently studied. NK cells need an antigen stimulation together with costimulatory signals such as OX40 or GITR. Adenosine receptors A2AR and A2BR both decrease proliferation of NK cells. Blockade of CD73 increases NK cell function. NK cells can also be stimulated by Fms-like tyrosine kinase3-ligand, GM-CSF, IL2, and IL15^[92]^. Many pulmonary adenocarcinomas express A2A receptors. If these receptors are inhibited, this results in impaired growth of tumor cells and carcinoma associated myofibroblasts. If a carcinoma is positively tested for high expression of A2A receptors, an inhibitory therapy might inhibit tumor cell growth^[93]^. Another effect of adenosine is in the interaction of tumor cells and platelets: Tumor cells interacting with platelets induce a release of adenosine, and this induces an opening of the endothelial barrier, subsequently allowing transendothelial migration of tumor cells. The primary mediator of this effect is the endothelial P2Y2 receptor, which is activated by ATP^[94]^. Another ATP-gated receptor P2X(7) is expressed in multiple malignant tumors. Prolonged ATP-mediated activation of P2X(7) leads to formation of pores, increases membrane permeability and eventually causes cell death. This is a paradoxon: The TME contains high levels of extracellular ATP, which can activate the P2X(7) and trigger cell death. However, P2X(7) expression is associated with enhanced cancer cell survival, proliferation and metastatic potential. The explanation might be within a non-pore functional P2X(7)^[95]^. ATP and activation of P2 receptors are also linked to amplification of TGF-β1 induced migration of lung cancer cells^[96,97]^. Exposure to high ATP concentration induces P2X(7) expression and is essential for tumor cell survival, as ATP is rapidly internalized by micropinocytosis and thus supply energy to the tumor cells. This opens the possibility of another line of therapy^[98,99]^.

## Tertiary lymph follicles

A new option was recently discussed for carcinomas of the immune-desert type. Tertiary lymph follicles (TLF) are formed in some carcinomas. In these TLFs, there is a T cell zone with CD3 expressing lymphocytes and dendritic cells (DC) expressing LAMP3, a follicular zone with CD20^+^ lymphocytes and mature DCs. This can be simulated by CXCL13 and IL17, which will result in the recruitment of lymphoid tissue. Bcells and M1 macrophages can substitute this effect. An interaction with stroma cells by lymphotoxin-α1β2 and binding to lymphotoxin receptor leads to the secretion of VEGFC by the stroma cells. This in turn induces the formation of high endothelial venules, which cells secrete VCAM1 or MADCAM1 [Figure 7B]. As a result, lymphocytes are recruited to this area. A form of therapy might be beneficial using a mixture of cytokines (analogs) to induce high endothelial venule formation and finally influx of cytotoxic lymphocytes^[100,101]^.

Another approach is the sequential combination of chemotherapy or radiotherapy, which can create TLFs, followed by the application of PDL1 therapy combined with either LAG3 or TIM3^[100,102]^.

## Prostaglandins

The inhibition of the prostaglandin system has always been a topic of discussion. Cyclooxygenase 2 (COX2) and PGE2 inhibit maturation of DC and activation of NK and T cells, and promotes differentiation of macrophages towards the M2 type. Inhibition of COX2 had shown some benefit in cancer therapy^[103]^.

## Design of cytotoxic lymphocytes and killer cells

A new way to target the immune system is the Car-technology (chimeric antigen receptor, Car). A fusion molecule is produced from the neoantigen with a fragment of the T cell receptor. This fusion molecule is expanded in patients’ lymphocytes. This was effectively applied in Bcell lymphomas. However, severe cytotoxicity (cytokine release syndrome) was seen when this therapy was applied in solid tumors^[104,105]^. A progress was made by combining Car T cell therapy with the gene editing system Crispr/Cas^[106]^. This field has expanded: instead of modified lymphocytes, the focus has shifted to natural killer (NK) cells and macrophages. These cells could also be modified to detect neoantigens from cancer cells and attack them. This therapy has much less toxicity, less cytokine release and less neurotoxicity^[107-110]^. NK cells could be harvested from pluripotent stem cells; in addition, macrophages could be polarized to M1 type.

## Phagocytosis and the immune system

A very recent focus was drawn to phagocytosis. Phagocytosis is essential for macrophages and antigen presenting cells. A neoantigen needs to be phagocytosed to be processed. Don’t-eat-me signals were detected, which support cancer development and progression. This don’t-eat-me signal involves CD24 binding to sialic acid binding IG-like lectin 10 (Siglec10). This signal blocks innate immune cells, especially macrophages. A blockade of the CD24-Siglec10 binding decreased tumor growth^[111]^. Thus, another option for immunotherapy of cancer has been explored. Interestingly this is not the one-and-only phagocytosis checkpoint: Feng *et al*.^[112]^ described in their review another phagocytosis checkpoint, namely the axis CD47-SIRP, another inhibitor of phagocytosis [Figure 7C]. On the other end, calreticulin promotes phagocytosis by macrophages. If the CD47-SIRPa axis is disrupted by a CD47 antibody, phagocytosis was increased and tumor progression inhibited. Under this treatment, an increase of M1 macrophages was observed. This treatment approach is also now tested in combination with PDL1.

Expression of CD47 has recently been reported in EGFR-mutated pulmonary adenocarcinoma cell lines. A treatment with an EGFR inhibitor combined with an CD47-blocking antibody induced downregulation of CD47 and increased phagocytosis by monocyte-derived dendritic cells (probably interdigitating dendritic cells)^[113]^. However, there are some limitations in this study: the results were based on cell cultures, focused especially on EGFR-mutated adenocarcinomas, and the dendritic cells were derived from blood monocytes, which do not reflect the dendritic cell population seen within NSCLC tissues^[67,114]^. Treatment trials have been installed, and first results seem promising. One study used intra-tumoral vaccination with autologous dendritic cells^[115]^ and resulted in an increase of anti-tumor response by T cell infiltration. An ongoing trial is focusing on patients with surgical resected stage II and III NSCLC, who were also postoperatively treated by chemotherapy; pulsed dendritic cells are infused into patients to install an anti-tumor immune reaction^[116]^. Another study focused on the ICOS/ICOSL system, which is expressed on cells of the innate immune system, especially on dendritic cells. Blockade of the ICOS-system might improve T cell mediated cytotoxicity against lung cancer^[117]^.

Bronchoalveolar lavage (BAL) is used for the evaluation of inflammatory diseases of the lung by washing out the lung lobes affected by an inflammatory/immune disease. Typing of lymphocytes allows for evaluation of disease activity, impact of therapy, and in few diseases also the diagnosis of the disease^[118-122]^. In cases of lung tumors, BAL is most often used to collect tumor cells from peripheral tumors not otherwise accessible. However, this tool might also be used to analyze the percentage of immune cells in lung lobes bearing lung carcinomas or molecules associated with immune reactions.

Response to immunotherapy cannot be assessed with any of the present-day markers. Neither PDL1, nor tumor mutational burden by itself can predict response with certainty. Expression of different modulators of immune responses such as CD73, CD38, CD24, CD47 and many more can be evaluated on tumor cells, immune cells, or as soluble markers in BAL.

Another approach recently tested by many investigators is to harvest circulating tumor cells together with platelets, lymphocytes, macrophages, and neutrophils and to study their interaction (for example by cDNA or proteomic analysis). In addition, organ cultures cultivated together with their stroma/microenvironment opens such an insight into the immune mechanisms acting in a given carcinoma^[123,124]^.

HLA types associated with response/resistance (HLA diversity) should be included into the tests, as well as the analysis of the TMA with infiltrating T-lymphocytes (Treg, T-CD8), neoantigen diversity, clonal/subclonal neoantigen diversity, *etc*.^[20,26]^. The landscape of tumor immunology is almost daily expanding, and we are only at the beginning of understanding the multitude of mechanisms which play a role in the tumor immune system interplay.

## Conclusion

We have discussed the PD1/PDL1 checkpoint inhibition and the mechanisms of resistance. We focused on cells of the specific and innate immune system and their role in immune tolerance, as well as possibilities to introduce new therapy options. We finally focused on the new field of soluble mediators involved in regulating the tumor-immune system interaction, and new therapy avenues. At the end, some thoughts on methods for better evaluating this tumor immune system interaction were discussed.

## Declarations

### Copyright

© The Author(s) 2020.
